# Rapid, Simple and Inexpensive Fabrication of Paper-Based Analytical Devices by Parafilm^®^ Hot Pressing

**DOI:** 10.3390/mi13010048

**Published:** 2021-12-29

**Authors:** Surasak Kasetsirikul, Kimberley Clack, Muhammad J. A. Shiddiky, Nam-Trung Nguyen

**Affiliations:** 1 Queensland Micro- and Nanotechnology Centre (QMNC), Griffith University, Nathan, QLD 4111, Australia; surasak.kasetsirikul@griffithuni.edu.au (S.K.); kimberley.clack@griffithuni.edu.au (K.C.); m.shiddiky@griffith.edu.au (M.J.A.S.); 2 School of Engineering and Built Environment (EBE), Griffith University, Nathan, QLD 4111, Australia; 3 School of Environment and Science (ESC), Griffith University, Nathan, QLD 4111, Australia

**Keywords:** paperfluidics, parafilm, paper-based analytical devices

## Abstract

Paper-based analytical devices have been substantially developed in recent decades. Many fabrication techniques for paper-based analytical devices have been demonstrated and reported. Herein, we report a relatively rapid, simple, and inexpensive method for fabricating paper-based analytical devices using parafilm hot pressing. We studied and optimized the effect of the key fabrication parameters, namely pressure, temperature, and pressing time. We discerned the optimal conditions, including a pressure of 3.8 MPa, temperature of 80 °C, and 3 min of pressing time, with the smallest hydrophobic barrier size (821 µm) being governed by laminate mask and parafilm dispersal from pressure and heat. Physical and biochemical properties were evaluated to substantiate the paper functionality for analytical devices. The wicking speed in the fabricated paper strips was slightly lower than that of non-processed paper, resulting from a reduced paper pore size after hot pressing. A colorimetric immunological assay was performed to demonstrate the protein binding capacity of the paper-based device after exposure to pressure and heat from the fabrication. Moreover, mixing in a two-dimensional paper-based device and flowing in a three-dimensional counterpart were thoroughly investigated, demonstrating that the paper devices from this fabrication process are potentially applicable as analytical devices for biomolecule detection. Fast, easy, and inexpensive parafilm hot press fabrication presents an opportunity for researchers to develop paper-based analytical devices in resource-limited environments.

## 1. Introduction

Paper-based analytical devices or paperfluidic devices have attracted enormous attention in the past few decades. They demonstrated the possibility of being cost-effective, biodegradable, and used as platforms for point-of-care diagnostic devices [1,2,3]. Following the emergence of paper-based microfluidic technologies established by the Whitesides group in 2007 [4], many studies indicated the resilience of paper-based devices in food industry, environmental science, and medical diagnostics [1,2,3,5,6]. The basic principle behind paper-based technology is making paper both hydrophobic and hydrophilic, so that flow and biological assay properties can be handled within a single device. The paper-based fabrication process can be classified into two main steps: (i) deposition of patterned hydrophobicity via photolithography [7,8], wax plotting [9], polymer or wax printing [10,11], and other deposition methods [12,13,14,15,16,17], and (ii) removing hydrophobic material to get the final pattern, such as inkjet etching [18], plasma etching [19], chemical wet etching [20], or laser treatment [21,22]. However, some fabrication methods involve harsh chemicals such as tetramethyl orthosilicate (TMOS), which may be left on the paper, and inhibit the biochemical reaction [20]. In addition, some of the fabrication steps require precise and sophisticated equipment such as laser treatment or UV/photolithography systems [1,6,23]. Some other fabrication methods are simple and inexpensive. However, they yield low resolution and low reproducibility [5,23,24,25].

To solve the issues concerning chemical usage and low reproducibility, alternative methods and materials are required. Parafilm, which is a thermoplastic made from wax and polyolefin, was commonly used in wet laboratories. Dunfield et al. reported in 2012 the first attempt to melt parafilm onto the paper by incorporating pressure and heat [26]. They suggested that polymer film can prevent the melted parafilm from getting into the paper as it provides a hydrophobic area. Nonetheless, there is no further and detailed study on the relation of fabrication parameters for analytical applications. Yu et al. fabricated a 2D and 3D paper-based analytical device with photolithography and embossing of parafilm [27]. However, this fabrication process required multiple steps and special equipment for lithography, which took around 30–40 min to make a device. Recently, Kim et al. studied the use of low-temperature ranges to infuse parafilm into the paper and laser ablation to remove parafilm infused paper for fluidic channels [22]. This method can create a microchannel with a width down to 150 µm, depending on the resolution of laser ablation. Nevertheless, this fabrication method required specific and delicate equipment. 

Despite the recent advancements in paper-based analytical devices, no previous studies have investigated the specific relationship between fabrication parameters, such as temperature, pressure and pressing time. Herein, we optimize these parameters and their relationship to develop a simple and inexpensive analytical device for detecting biological targets via the colorimetric technique. To the best of our knowledge, this proof-of-concept analytical device is to date the only device prepared by the parafilm hot pressing method. Parafilm hot pressing allows for the penetration of parafilm into the paper and represents a rapid, simple, and inexpensive method for fabricating a paper-based device. The technique requires only polymer film, parafilm, paper, and a hot press to fabricate a paper-based device. The entire process takes only 5–10 min for two steps: cutting and pressing, resulting in a cost of less than 0.01 USD per device. We studied the effect of temperature (60–90 °C), pressure (2.5–5.1 MPa), and pressing time (1–5 min) to obtain the optimal fabrication parameters. Following the fabrication, we demonstrated the functionality of the paper-based devices with wicking, colorimetric immunological assays, 2D diffusive mixing and a 3D paperfluidic system.

## 2. Materials and Methods

### 2.1. Materials and Chemicals

The material used for the devices in our study is chromatography filter paper grade 1 (CHR, WHA3001861, Whatman, Kent, UK). Parafilm (Bemis Company, Birmingham, UK) was used to make the paper locally hydrophobic. A gloss laminate pouch with 80-µm thickness serves as a patterning mask for the front and back support during the fabrication process. For the colorimetric immunological assay, rabbit monoclonal anti-human CD9 (rabbit anti-CD9, ab92726, Abcam, Bristol, UK) and goat anti-rabbit IgG (anti-rabbit IgG, ab6721, Abcam, Bristol, UK) were conjugated with HRP using an HRP-conjugation kit (ab102890, Abcam, Bristol, UK). 3,3′,5,5′-tetramethylbenzidine substrate solution (TMB, 002023, Thermo Fisher Scientific, Waltham, MA, USA) was used to employ the colorimetric immunological assay, facilitated by the HRP/TMB reaction. Bovine Serum Albumin (BSA, A1595, Sigma Aldrich, Burlington, MA, USA) was used to block the sensor surface to minimize the background signal due to non-specific binding. For mixing applications, 0.1 M sodium hydroxide solution (SL178, Chem-Supply, VIC, Australia) and 1% phenolphthalein in ethanol (FE0496G100, Scharlau, Barcelona, Spain) were used as a mixing indicator. Four food colour dyes (Queens Fine Foods, QLD, Australia) consisting of red, yellow, green, and blue were mixed with DI water (MilliQ, Merck, NY, USA) to obtain a 10% by volume ratio to illustrate the 3D paperfluidic channels.

### 2.2. Fabrication Parameter Study

**Figure 1:**
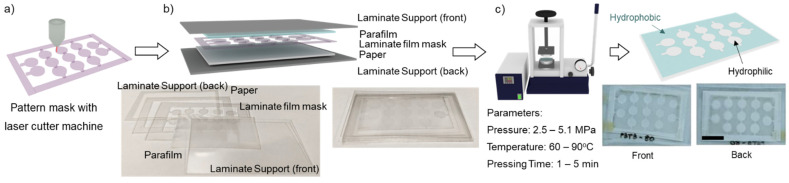
Schematic diagram of the fabrication process. (**a**) Pattern laminate films were cut with laser cutter machine; (**b**) The laminate supports, parafilm, patterning laminate film, and paper were stacked and covered with aluminium foil orderly (aluminium foil is not shown); (**c**) The stacked paper was placed in a hot press machine to locally determine hydrophobic to the paper. Scale bar is 1 cm.

Parafilm is a translucent, flexible film composed of waxes and polyolefin. Parafilm becomes soft and adhesive at a temperature between 54–66 °C [27]. Parafilm can be infused into the paper matrix by applying a certain pressure and temperature [22,26]. Parafilm can be melted and permeated into paper through a patterning mask. The laminate film was designed by CorelDraw software (CorelDRAW2019, Corel Corporation Inc, Ottawa, ON, Canada) and cut by a laser engraving machine (Rayjet 50 Laser Engraver, Trotec Laser, Wels, Austria). The size of the laser spot is 100 μm as given by the manufacturer. CHR (4.0 cm × 2.5 cm) was prepared and stacked with parafilm (3.5 cm × 2.0 cm), laminate film masks (working area of 3.5 cm × 2.0 cm and outer frame of 4.5 cm × 3.0 cm), and supports (4.5 cm × 3.0 cm), Figure 1. Subsequently, this paper stack was covered with an aluminium foil and placed into a hot press machine (Specac, London, UK). To optimize the fabrication parameters for paper-based analytical devices, the permeation of parafilm into the paper by combining the three parameters: pressure (2.5–5.1 MPa), temperature (60–90 °C), and pressing time (1–5 min). We investigated the permeation of parafilm into the paper to determine the optimal fabrication parameters. Briefly, images of the treated papers were imported and processed by a MATLAB script. The optimal condition must show full permeation of the parafilm in front and back side of the paper and the size of the hydrophilic area should approach the designed mask, which is a circle with a 5-mm diameter. A red colour dye was used to visualize and estimate the size of the hydrophilic area. The error bars applied to the measured data were two times of standard deviation.

### 2.3. Hydrophobic Barrier Resolution

**Figure 2:**
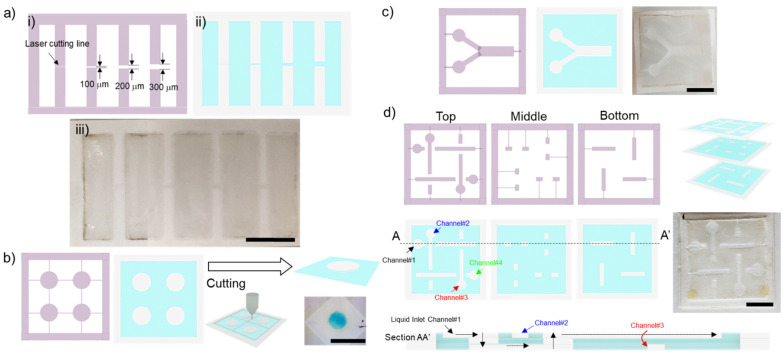
Laminate film for patterning masks with the laser cutting machine: (**a**) (**i**) pattern for investigating the resolution of the hydrophobic barrier (**ii**) the proposed pattern with gaps ranging from 100 to 300 μm after fabrication and (**iii**) the paper-based device after fabrication; (**b**) Pattern for the paper-based colorimetric sandwich immunological assay; (**c**) pattern for diffusive mixing in the 2D analytical paper-based device; (**d**) pattern of layers for constructing a 3D paperfluidic device and cross-sectional view of the 3D paper channel. Blue colour indicates the hydrophobic area, and white colour indicates the hydrophilic area. Scale bar is 1 cm.

The paper channel was patterned and determined by the laminate pattern mask, depending on the resolution of the laser engraving machine. The laminate film was cut with the single line to characterise the actual size of the laser cut line, Figure 2a. The smallest feature of the mask was determined by the size of a single cut line. We first investigated the resolution of the hydrophobic barrier after transferring the parafilm into the paper. The mask was designed to create a hydrophobic barrier between two straight channels (Figure 2a). The gaps were designed to be 100, 200 and 300 µm. Two dye colours were used to visualise the straight channels and to evaluate the quality of the hydrophobic barrier.

### 2.4. Physical and Biochemical Properties of the Paper

#### 2.4.1. Flow Characteristic

Pressure and heat generated during the fabrication process may affect the wicking behaviour in the paper strips, so we investigated the flowing characteristics between fabricated and original paper strips. The paper strip was prepared with a laser cutting machine, with a width of 2 mm, 4 mm, and 6 mm. The capillary rise experiment setup was adapted from our previous study [28]. Briefly, the paper was vertically positioned with a customized acrylic stand. The dye solution was loaded into the liquid reservoir below the paper strip. The fluid wicking up to the paper strip was recorded, processed, and quantified using MATLAB to determine the relationship between the distance of the liquid front and time.

#### 2.4.2. Colorimetric Sandwich Immunological Assay

The colorimetric sandwich immunological assay demonstrates that heat and pressure from the fabrication process are compatible with the protein binding capacity on the paper matrix. The laminate film was cut into a 5-mm diameter patch with support, Figure 2b. Subsequently, the paper was cut into individual devices. We employed the HRP/TMB reaction to illustrate the colorimetric assay. In the presence of HRP, TMB is oxidized, changing from colourless to a blue colour complex. Briefly, the paper was coated with 5 µL of 0.1 mg/mL rabbit anti-human CD9, with subsequent blocking with 2% BSA. A total of 3 µL of 1 µg/mL of goat anti-rabbit IgG conjugated with HRP was added to make a sandwich immunoassay. We performed an analytical assay with and without coating rabbit anti-human CD9. The results were recorded with the setup adapted from our previous study at the 20th min [29].

### 2.5. 2D and 3D Paperfluidic Devices

#### 2.5.1. Diffusive Mixing in the Two-Dimensional Paperfluidic Device

We investigated diffusive mixing on an open-channel two-dimensional (2D) paper-based device. The laminate film was cut to have two inlets and a straight channel, Figure 2c. The hydrophilic channel on the paper is defined by the patterning mask. For characterization of the mixing process, 10 µL of 0.1 M sodium hydroxide and 10 µL of phenolphthalein solution were dropped on the left and right inlets, respectively. After mixing with the alkaline solution, the colour of phenolphthalein changes from colourless to dark pink. The dark pink colour developed in the straight channel confirms the diffusive mixing capability in the paper-based device.

#### 2.5.2. Flow in a Three-Dimensional Paperfluidic Device

We also demonstrated fluid flow in the three-dimensional (3D) paper-based device. This paper device was composed as a stack of three hot-press papers, Figure 2d. Each paper layer was fabricated with the optimized conditions and then assembled together using solvent-free glue stick. The different dye solutions were wicked through the straight channel between layers and demonstrate the 3D flow configuration. The food dye is expected to maintain its colour at the end of the channel because the patterned hydrophilic channels do not intersect and cross-contaminate each other. 

### 2.6. Data Acquisition and Quantification

Images of the device were captured by a digital camera (PowerShotA60, Canon, Tokyo, Japan) and evaluated with ImageJ (NIH, Bethesda, MD, USA). For colorimetric quantification, the image was imported and processed with MATLAB. First, the region of interest (ROI) was cropped. Next, the ROI pixel values were split into red, green and blue channels. Next, the mean grey value of *RGB_value_* was calculated as:

(1) $$RGB_{value}=\sqrt{{\left(R-R_{0}\right)}^{2}+{\left(G-G_{0}\right)}^{2}+{\left(B-B_{0}\right)}^{2}}$$

where *R*, *G*, and *B* are the mean grey values from images for red, green, and blue channels, respectively. The mean grey values from the white background are defined as *R*_0_, *G*_0_ and *B*_0,_ which are red, green, and blue channel values, respectively. 

## 3. Result and Discussion

### 3.1. Optimization of Fabrication Parameters

#### 3.1.1. Effect of Temperature

**Figure 3:**
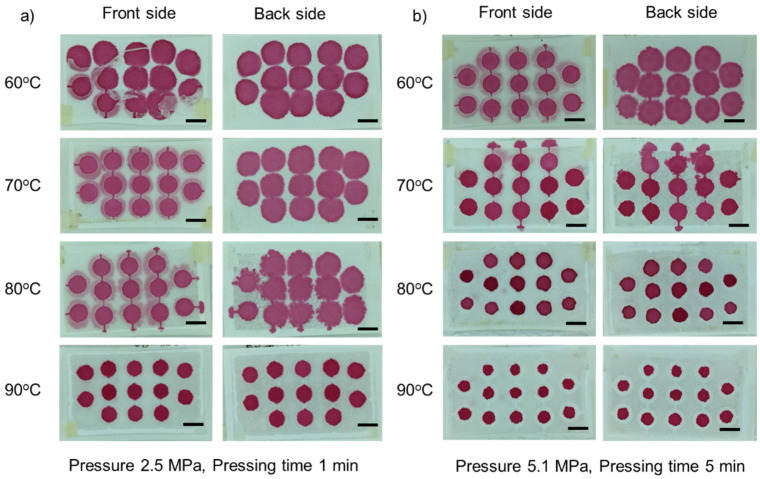
Effect of temperature: (**a**) under a pressure of 2.5 MPa and 1 min pressing time; (**b**) under a pressure of 5.1 MPa and 5 min of pressing time. The temperature was varied from 60 to 90 °C (left column shows the front side, right column shows the back side). Scale bar is 5 mm.

The temperature was varied over the range of 60 °C to 90 °C to optimize the temperature during fabrication. Maintaining the pressure at 2.5 MPa and 1 min of pressing time, we found that the parafilm can be melted at a relatively high temperature and can penetrate through the paper, Figure 3a. We observed that at 60 °C, parafilm could not adhere to the paper. Parafilm can permeate into the paper at temperatures of 70 °C and 80 °C, but it does not fully penetrate the paper as observed from the back, where the parafilm cannot be seen. At a temperature of 90 °C, parafilm was fully permeated into the paper. Nevertheless, if the pressure and pressing time are increased, a high temperature can result in over-permeation, Figure 3b. Providing the pressure of 5.1 MPa and 5 min of pressing time, parafilm at 60 °C still cannot permeate into the paper even at a higher pressure and longer pressing time. At temperatures of more than 80 °C, parafilm can fully penetrate the paper matrix, but the working area defined by the patterning mask became smaller due to overflow. In conclusion, the temperature in hot press fabrication plays a role in melting the parafilm.

#### 3.1.2. Effect of Pressure

**Figure 4:**
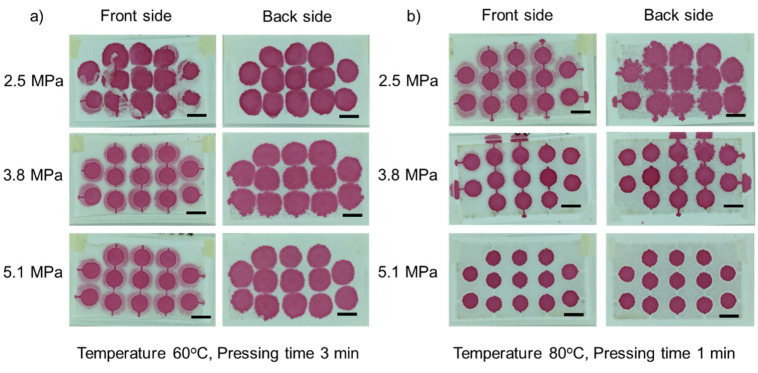
Effect of pressure: (**a**) under a temperature of 60 °C and 3 min of pressing time; (**b**) under a temperature of 80 °C and 3 min of pressing time. The pressure was varied from 2.5–5.1 MPa in the row (left column shows the front side, right column shows the back side). Scale bar is 5 mm.

Pressure was varied in the range of 2.5–5.1 MPa to investigate the role of pressure in the fabrication process. We observed that under 3 min of pressing time, a relatively low temperature of 60 °C and an applied pressure of 2.5 and 3.8 MPa, Figure 4a, parafilm rarely penetrates into the paper, as it cannot be observed at the backside of the paper. With an applied pressure of 5.1 MPa, parafilm slightly permeated through to the back of the device. In contrast, a temperature of 80 °C and 3 min of pressing time enable parafilm to permeate into the paper under applied pressures of 2.5 and 3.8 MPa, Figure 4b. However, an applied pressure of 5.1 MPa resulted in a slight overflow of parafilm on both the front and back sides. Thus, the pressure plays a significant role in pushing melted parafilm into the paper matrix. 

#### 3.1.3. Effect of Pressing Time

**Figure 5:**
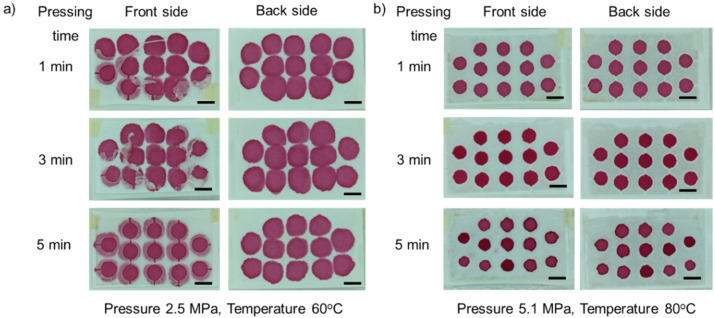
Effect of pressing time: (**a**) under a temperature of 60 °C and a pressure of 2.5 MPa; (**b**) under a temperature of 80 °C and a pressure at 5.1 MPa. The pressing time was varied from 1 to 5 min (left column shows the front side, right column shows the back side). Scale bar is 5 mm.

We investigated the effect of pressing time used in this fabrication in the range of 1–5 min. At a temperature of 60 °C, partially melted parafilm still adheres to the paper under an applied pressure of 2.5 MPa. Even though parafilm does not fully penetrate under this condition, 5 min of pressing time are sufficient to push parafilm into the paper matrix, Figure 5a. While a temperature of 80 °C successfully melts and pushes parafilm through the paper for all pressing times from 1 to 5 min, Figure 5b. However, a longer pressing time can cause parafilm to over-penetrate and to seep into the pattern. Clearly, pressing time also plays a significant role in assisting parafilm to penetrate the paper matrix. 

**Figure 6:**
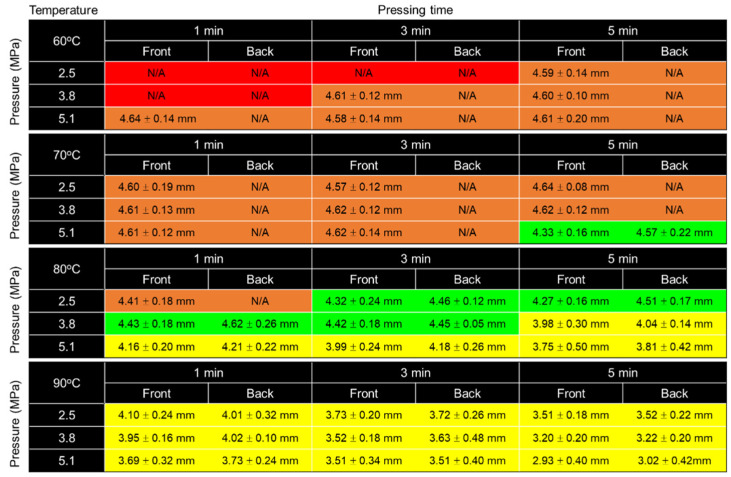
The colour-coded evaluation matrix of fabrication parameters and the diameter size of circular hydrophilic area after the fabrication. N/A denotes that actual circular size of diameter cannot be measured due to incomplete parafilm permeation. At least 3 samples were measured. Colour codes: red—no parafilm on both sides, orange—parafilm has not fully permeated, green—parafilm can be seen on both sides, and yellow—over-penetration.

With the varying temperature, pressure and pressing time, the optimal fabrication parameters were selected by two criteria: (i) the parafilm must be observed by front and back sides of the paper and (ii) the size is the closest to that of the designed pattern. Figure 6 represents the evaluation result, which is labelled with four different colours; red for no parafilm on both sides, orange for parafilm that has not fully permeated, green for parafilm that can be seen on both sides and the closest size to the designed circular diameter, and yellow for over-penetration. Figure 6 shows that the optimal conditions for the paper-based analytical device used in this study are the temperature at 80 °C, pressure at 2.5–3.8 MPa, and 3 min of pressing time. In addition, for the manual hot press machine, pressure can be reduced over time due to the restoration of the sample between two pressing plates. Therefore, a pressure of 2.5–3.8 MPa can successfully push parafilm into the matrix and does not cause over-penetration within 3 min of pressing time and at a temperature of 80 °C.

### 3.2. Hydrophobic Barrier Resolution

**Figure 7:**
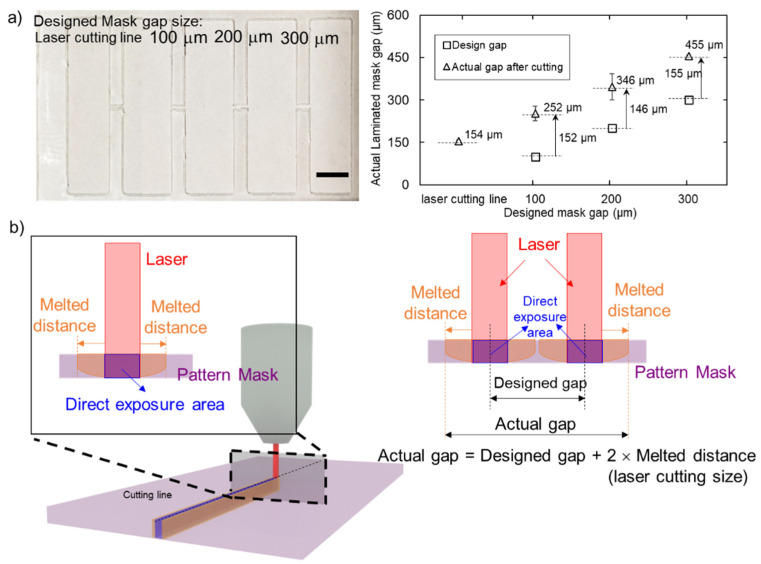
Laminate mask resolution: (**a**) the actual gap from laminated mask which was increased approximately 150 µm in all designs, resulting from the laser point size and local laminated mask melting during cutting process; (**b**) the schematic diagram for laser cutting, showing that the actual gap originating from the laser cutting process is always larger than the designed one. Scale bar is 5 mm.

The laminate film mask determines the resolution of the fabrication process. As a laser cutting machine uses heat to melt and cut the materials, the smallest size resulting from the cutting mask is the size of the laser point. Figure 7a shows the actual gap of the laminate mask from the design. The actual gap size of a single line was 154 ± 5 µm. In addition, for 100 µm, 200 µm, and 300 µm designed masks, the actual gaps were 252 ± 25 µm, 346 ± 46 µm, and 455 ± 3 µm, respectively. Interestingly, the actual gap made with a single cut is 154 µm, which is larger than laser spot size of 100 µm. Moreover, we found a similar trend for 100 µm, 200 µm and 300 µm designed gaps. An increase of 150 µm from the designed value results from the cut of a single line, Figure 7b.

**Figure 8:**
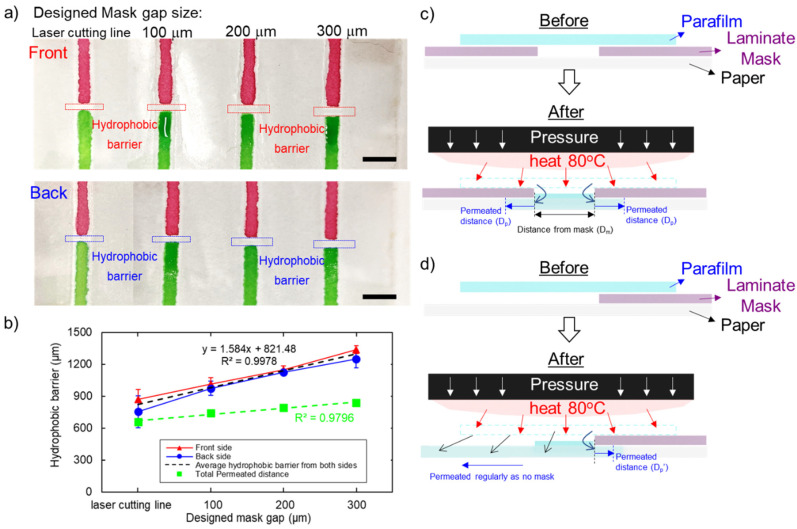
Resolution of hydrophobic barrier: (**a**) hydrophobic barrier is tested with food dye colours; (**b**) a linear relationship between the final hydrophobic barrier with the designed mask, indicating the smallest hydrophobic barrier size is 821 µm; (**c**) parafilm melting into the paper through the gap of the mask; (**d**) parafilm melting into the paper through the open space of the mask. Both schematic diagrams show that the melting could be dictated by the gap in the mask (D_m_) and permeated distance (D_p_). Scale bar shown is 5 mm.

With the selected fabrication temperature of 80 °C, applied pressure at 3.8 MPa and 3 min of pressing time, parafilm can penetrate the paper to form a hydrophobic area. Figure 8a illustrates that the hydrophobic barrier on the front side (red markers) is slightly larger than on the backside (blue markers). However, the data between the front and back sides under all conditions are not significantly different (*p* < 0.05). Therefore, we consider that the hydrophobic barrier size used for analysis can be the average of those from the front and back sides. The average hydrophobic barrier size was 813 ± 182 µm, 994 ± 87 µm, 1137 ± 47 µm and 1293 ± 89 µm for the actual average mask gap of 154 µm, 252 µm, 346 µm, and 455 µm, respectively. The parafilm can be melted and dispersed into the paper, which is larger than the actual gap. We found that parafilm dispersal distance results from two parameters which are the patterned mask size (D_m_) and permeation distance (D_p_). Figure 8b shows that permeation distance (plotted as green markers) is 659 µm, 741 µm, 790 µm, and 838 µm for the actual gap of 154 µm, 252 µm, 346 µm, and 455 µm, respectively, which linearly increased (green dashed line with *R*^2^ = 0.9796). The increased permeation of parafilm may result from the presence of additional parafilm between the gap, penetrating into the paper.

As a result, we found a linear relationship between the designed gap (in micrometres for the *x*-axis and hydrophobic barrier size in micrometres for the *y*-axis) with the trend line of *y* = 1.584*x* + 821.48 (*R*^2^ = 0.9978) as shown in Figure 8b. This equation can be used to estimate the hydrophobic barrier size corresponding to the designed pattern. This relationship includes the effect of the laminated mask and the permeation of parafilm in the fabrication. The smallest hydrophobic barrier structure can be formed with this method is 821 µm, which is a similar result for the case of a laser cut line in the designed mask.

In addition, D_p_ was determined from the optimisation experiments. Figure 6 indicates the actual diameter under the optimised condition (temperature 80 °C, pressure 3.8 MPa, pressing time 3 min) was approximately 4.4 mm. The smaller size compared to the designed 5-mm diameter is possibly caused by the permeation effect. However, D_p_ may vary with different masks. The permeation in the local area depends on the ratios of parafilm to paper as shown in Figure 8c,d [22,30]. 

A hydrophobic barrier is formed on paper by loading hydrophobic materials into the paper matrix. Therefore, parameters such as the amount of hydrophobic material and physical properties of the paper such as thickness, pore size need to be considered. More wax can permeate into the paper if a large amount of wax is used [30]. Moreover, the pore size and thickness of the paper also affect the permeation capability. For example, polyvinylidene difluoride membrane (PVDF) requires more incubation time to allow wax to fully permeate into the membrane as compared to Whatman filter paper [27]. The smallest hydrophobic barrier created with our method is relatively larger than other methods. To further decrease the barrier size, we proposed the following methods. First, papers with smaller pore size such as mixed cellulose ester membrane (MCE) can reduce the spread of the wax and increase the hydrophobic area [27,30]. Second, we may reduce the amount of parafilm to limit the excessive permeation into the hydrophilic area [27,30]. Third, the mask resolution needs to be increased to allow less parafilm to pass through the gap.

### 3.3. Physical and Biochemical Properties of the Paper

#### 3.3.1. Flowing Characteristics

**Figure 9:**
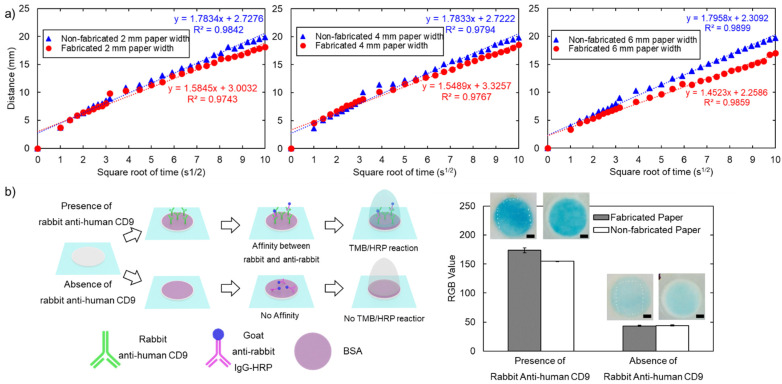
Physical and biochemical properties of the paper: (**a**) flow characteristics resulting from 2-mm, 4-mm and 6-mm paper strips. A blue marker for original paper and a red marker for fabricated paper; (**b**) schematic diagram for colorimetric immunological assay in the presence and absence of rabbit anti-human CD9 with the bar diagram for *RGB_value_* quantified at the 20th minute of the TMB/HRP assay with MATLAB between fabricated (grey bar) and non-fabricated paper (white bar). Error bars represent the standard deviation from the average of three replicates (*n = 3)*. Scale bar is 1 mm.

We investigated the wicking mechanism to evaluate the physical properties of the paper following the fabrication process. We observed wicking in the paper strip and plotted the relationship between the distance of the liquid front versus the square root of time. Figure 9a indicates that the fabricated paper results in a slightly smaller slope than the original paper (1.45–1.58 for fabricated paper vs. 1.78–1.79 for original paper). According to the Washburn relationship, the slope between the distance of the liquid front and the square root of time is proportional to the pore size of the paper matrix [31]. A lower slope may originate from the smaller pore size resulting from pressure in the fabrication process [32]. However, the wicking mechanism still follows the conventional Washburn relationship with R-square (0.974–0.986 for fabricated paper vs. 0.980–0.990 for original paper). Moreover, a lower wicking speed may also enhance sensitivity, allowing more time for a reaction to occur and better results. Consequently, longer fluid wicking in an open environment may encounter evaporation issues [33,34]. Overall, the pressure and heat from the fabrication process may affect the wicking mechanism by slightly decreasing the wicking speed, but it does not break the Washburn relationship.

#### 3.3.2. Colorimetric Sandwich Immunological Assay

We performed a colorimetric sandwich immunological assay to evaluate protein binding on the paper matrix after hot pressing fabrication, Figure 9b. The image was processed and quantified as per the bar diagram shown in Figure 9b. The result shows that in the presence of rabbit anti-human CD9, goat anti-rabbit IgG conjugated with HRP would be captured resulting from the immunoaffinity interaction, subsequently reacting with TMB, resulting in the development of a blue-coloured charge transfer complex due to HRP/TMB reaction. In contrast, in the absence of rabbit anti-human CD9, goat anti-rabbit IgG conjugated with HRP would be absent due to the lack of an immunoaffinity interaction, remaining as a colourless solution. However, a slight blue colour may be present due to non-specific adsorption. Blue colour development at the 20th minute in the presence of rabbit anti-human CD9 (grey bar in Figure 9b) was slightly higher than that of original paper (white bar in Figure 8b). Under the same conditions, TMB solution remains on the fabricated paper as seen by the light reflection in the light box. In contrast, on original paper, there is no leftover liquid form of TMB solution. Heat and pressure resulting from fabrication may result from the smaller pore size of the paper as a result of more reaction time leading to more blue intensity [34]. This observation corresponds to the wicking experiment in the previous section showing that wicking in the fabricated paper strip is slightly slower than in original paper. As demonstrated by the TMB/HRP reaction, the immunological assay can be performed with the hot-press fabricated paper.

### 3.4. 2D and 3D Paperfluidic Applications

**Figure 10:**
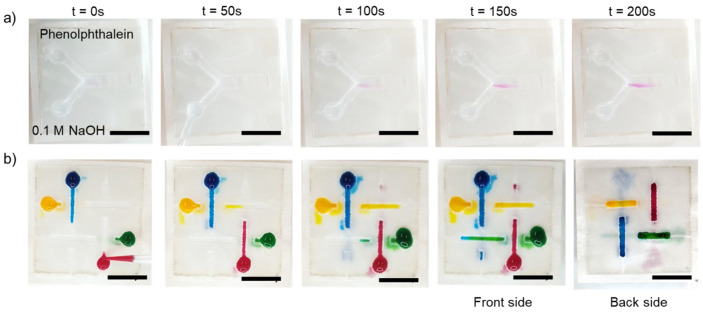
Analytical assay test: (**a**) diffusive mixing between 0.1 M NaOH and phenolphthalein pH indicator in the 2D analytical paper-based device; (**b**) flow in the 3D configuration paper-based device showing that there was no mixture of colour as shown in the final front and back side of the paper device. Scale bar is 5 mm.

We performed mixing experiments in 2D paperfluidic devices and 3D paperfluidic channels to demonstrate the 2D and 3D paperfluidic applications. For diffusive mixing in 2D paperfluidic devices, 0.1 M NaOH and phenolphthalein pH indicator were dropped at the inlets for interaction in the straight channel shown in Figure 10a. The colour changed from colourless to pink, showing that the chemical reaction can take place in over 0 to 200 s in our paper device. The paper-based device made with our method is compatible with diffusive mixing. Moreover, we implemented a 3D paperfluidic channel, as shown in Figure 10b. The liquid can independently flow in certain channels without mixing with other channels as the same colour can be seen from its reservoir to the end of the channel. As a result, the paper-based device (fabricated layer-by-layer and assembled to create 3D paperfluidics) was successfully demonstrated. As such, parafilm hot pressing fabrication has a great potential to be applicable to more complex analytical applications in both 2D and 3D configurations.

## 4. Conclusions

In conclusion, we have successfully fabricated paper-based analytical devices using parafilm hot pressing. The fabrication process for paper-based devices reported here offers (i) speed with an entire process time of less than 5 min, (ii) simplicity as only two steps are needed: cutting the mask with a laser cutting machine and hot pressing, and (iii) low cost as the total fabrication cost being less than 0.01 USD per device, Table 1. We studied the effect of three fabrication parameters: pressure, temperature, and pressing time. The optimized conditions for the fabrication include pressure of 3.8 MPa, temperature of 80 °C, and pressing time of 3 min, with the determination of the equation of the approximate hydrophobic barrier size from the design of the mask gap. The smallest hydrophobic barrier resulting from the laminated mask and melting parafilm in the fabrication is 821 µm. However, the hydrophobic barrier and the resolution of the paper-based devices can be further reduced by selecting paper with smaller pore size, less parafilm to prevent excessive permeation, and improving mask resolution to obtain a smaller gap. Moreover, we also investigated the physical and biochemical properties of the paper-based device after the fabrication to affirm the functionality of the paper for analytical devices. In addition, we successfully demonstrated 2D diffusive mixing and 3D paperfluidic applications. We believe that parafilm hot pressing techniques have the potential for scaling up in mass production due to its rapid, simple, and inexpensive nature, and offer an alternative method for researchers to develop proof-of-concept paper-based analytical devices in low-resource settings.

## Figures and Tables

**Table 1:** Comparison of paper-based analytical devices fabrication techniques.

**Fabrication Process**	**Operation Time**	**Hydrophobic Barrier**	**Advantages**	**Disadvantages**	**Ref.**
Photolithography	40 min	~250 μm	Variety of patterns	Exposed to polymers and solvent, Expensive equipment and reagents	[4,7]
Plotting	1 h	~250 μm	Low-cost consumables, not exposed to harsh chemicals	Need customized plotter, Inconsistent control of hydrophobic barrier formation	[5,9]
Cutting	1–3 min	700 μm	Not exposed to harsh chemicals	Need hydrophobic substrates or cases to operate	[35]
Plasma Etching	1 h	<1500 μm	Low-cost consumables	Exposed to polymers and solvent, single-use mask	[19]
Wet etching	~3 h	<1000 μm	Low-cost consumables	Exposed to harsh chemicals	[20]
Laser etching	2 m/s (depending on the pattern)	600 μm	Selective modification	Require strong hydrophobic reagents	[21]
Inkjet etching	~2 h 30 m	>150 μm	Precise controlled location	Exposed to polymers and solvents, require customized printers	[18]
Inkjet printing	5–15 min	300–550 μm	Low-cost thermal inkjet printers	Require formulated ink and customized printers	[11,36]
Flexography printing	5–10 s	1000 μm	Well suited for mass production	Require expensive and modified equipment	[12]
Wax printing	5–10 min	100 μm	Rapid, simple process, mass production	Require customized printer, extra heating steps, and rough channel edge.	[11,37]
Wax dipping	<1 min	Depend on iron mold	Low-cost and simple process	Batch-to-batch variation	[14,38]
Wax screen printing	<5 min	500–1300 μm	Low-cost and simple process (0.3 USD/100 cm^2^)	Require patterning mesh, low resolution	[13,39]
Vapor deposition	~1 h 30 m	2500–3500 μm	Complex patterns	Require expensive equipment	[40,41]
Stamping	<1 min	>950 μm (Depend on stamp)	Low-cost and simple process	Batch-to-batch variation, resolution depends on the stamp	[42,43]
3D printing	~2 h	400–500 μm	Variety of patterns	Resolution on 3D printers and printing materials	[44,45]
Spraying	<5 min	<1000 μm (Depend on masks)	Rapid and simple process	Non-uniformity on spraying	[46,47]
Lithography and embossing	~40 m	~150 μm	High resolution	Unsuitable for mass production	[10]
**Parafilm Hot pressing**	**<5 min**	**>800 μm** **(Depend on masks)**	**Low cost (0.3 USD/100 cm^2^ paper), rapid, and simple process**	**Low resolution depending on the mask resolution**	
